# Metric-guided Image Reconstruction Bounds via Conformal Prediction

**Published:** 2024-04-23

**Authors:** Matt Y. Cheung, Tucker J. Netherton, Laurence E. Court, Ashok Veeraraghavan, Guha Balakrishnan

**Affiliations:** 1 Department of Electrical and Computer Engineering, Rice University; 2 Department of Radiation Physics, The University of Texas MD Anderson Cancer Center

**Keywords:** Uncertainty, Conformal Prediction, Inverse problems

## Abstract

Recent advancements in machine learning have led to novel imaging systems and algorithms that address ill-posed problems. Assessing their trustworthiness and understanding how to deploy them safely at test time remains an important and open problem. We propose a method that leverages conformal prediction to retrieve upper/lower bounds and statistical inliers/outliers of reconstructions based on the prediction intervals of downstream metrics. We apply our method to sparse-view CT for downstream radiotherapy planning and show 1) that metric-guided bounds have valid coverage for downstream metrics while conventional pixel-wise bounds do not and 2) anatomical differences of upper/lower bounds between metric-guided and pixel-wise methods. Our work paves the way for more meaningful reconstruction bounds. Code available at https://github.com/matthewyccheung/conformal-metric.

## 1 Introduction

Recent advancements in machine learning have led to novel imaging systems and algorithms that address ill-posed problems. Traditionally, image reconstruction evaluation relies on common image quality metrics such as PSNR, SSIM, FID, and Dice scores of segmentations on reconstructed images. While these metrics provide a heuristic to gauge the overall model uncertainty in reconstruction during evaluation, they do not provide uncertainties and guarantees at test time, and do not link reconstruction quality to uncertainties in downstream applications.

Conformal prediction (CP) provides distribution-free, valid, and calibrated prediction intervals at test time [32,10,29,2]. The idea is to use residuals from a calibration dataset to infer uncertainty in future test datasets [32,10,29,2]. For regression tasks, this uncertainty is given as a prediction interval [32,10,29,2]. The application of CP to image reconstruction has been relatively limited. This is a difficult problem because quantiles in higher dimensional data are M-quantiles, meaning they have infinite solutions and only have unique solutions when a direction is specified [6,7]. How do we pick such a direction? The conventional (pixel-wise) method is to pick the direction where all pixels are independent [12,16,9]. The upper and lower bounds of the estimated image can be calibrated based on pixel intensity [3,14]. While these pixel-wise prediction intervals are easy to interpret, they do not consider spatial correlations and may lead to large interval lengths [4]. The upper and lower bounds can also be calibrated in the direction of principal components [4]. While using principal components considers spatial correlations, it does not capture meaningful and practical uncertainty for downstream processes and is prohibitively costly to compute for large images. Furthermore, both methods provide upper and lower bounds not sampled from the learned manifold, yielding implausible images. A reasonable answer is to calibrate the upper and lower bounds in the direction of semantic features [27]. However, this method requires training a generative model with disentangled latent spaces.

We argue that bounds should be computed in the direction of downstream metrics for more reliable downstream performance. We propose “Metric-guided Image Reconstruction Bounds” that leverages CP to form valid prediction intervals of reconstructions in the direction of downstream metrics and retrieve reconstructions 1) closest to the upper and lower bounds, 2) contained in the bounds (statistical inliers) and 3) outside the bounds (statistical outliers). Our method takes spatial correlations into account and produces plausible reconstructions from the learned manifold. We show that our method provides valid coverage for downstream tasks while the conventional pixel-wise method does not and the upper/lower bounds between methods are anatomically different.

We demonstrate our method on sparse-view computed tomography (sv-CT) and downstream radiotherapy planning. Reconstruction is highly accurate for CT machines and uses sophisticated detectors and algorithms to obtain sub-millimeter spatial resolution. CT downtime significantly impacts the availability of radiotherapy planning in low-resource settings [11]. A low-cost device with cone-beam geometry could be manufactured and used to increase access to radiotherapy planning and other therapeutic use cases. Individualized radiotherapy plans are made from CT scans and specify localized doses to a target treatment volume (i.e. breast, prostate). We use downstream clinical application metrics from radiotherapy planning to retrieve reconstructions.

## 2 Method

**Fig.1.**
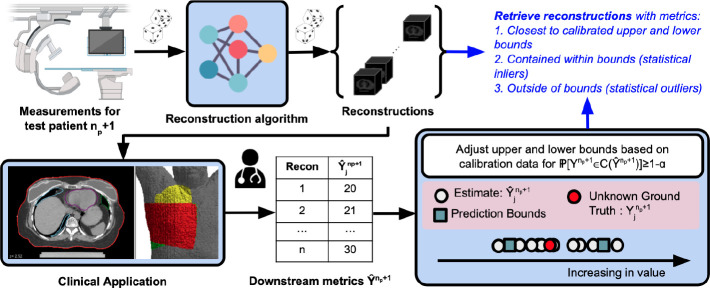
Overview of our approach. Assume probabilistic measurement and reconstruction processes, $n_{p}$ patients for calibration, and 1 patient for testing. For test patient $n_{p}+1$ with unknown ground truth reconstruction and metric, 1) acquire measurements, 2) attain a set of reconstructions, 3) extract downstream metrics, 4) adjust upper and lower bounds of metric based on a calibration procedure, and 5) retrieve reconstructions with metrics closest to the calibrated upper and lower bounds, contained within bounds (statistical inliers), and outside of bounds (statistical outliers).

We consider a 3-D reconstruction setting for a downstream application with a chosen downstream metric.^3^ The measurement and reconstruction algorithms are assumed to be probabilistic. We follow the split conformal prediction procedure [26,32,2,29] by using $n_{p}$ patients for the calibration dataset and 1 test patient $n_{p}+1$ with unknown ground truth volume and metric as the test dataset. For each patient i in the calibration dataset, we reconstruct a set of volumes of size ${\overset{ˆ}{V}}^{i}={\left\{{\overset{ˆ}{V}}_{j}^{i}\right\}}_{j=1}^{n_{r}}$ of size $n_{r}$. Each patient’s reconstructed volumes are used to attain a set of estimated metrics ${\overset{ˆ}{Y}}^{i}={\left\{{\overset{ˆ}{Y}}_{j}^{i}\right\}}_{j=1}^{n_{r}}$. Each patient’s ground truth volume is used to attain a ground truth metric $Y^{i}$. For the test patient, we reconstruct a set of volumes ${\overset{ˆ}{V}}^{n_{p}+1}={\left\{{\overset{ˆ}{V}}_{j}^{n_{p}+1}\right\}}_{j=1}^{n_{r}}$ and estimate metrics ${\overset{ˆ}{Y}}^{n_{p}+1}={\left\{{\overset{ˆ}{Y}}_{j}^{n_{p}+1}\right\}}_{j=1}^{n_{r}}$.^4^ Assuming $\left({\overset{ˆ}{Y}}^{i},Y^{i}\right)$ for $i=1,\ldots ,n_{p}+1$ are exchangeable, we leverage Conformalized Quantile Regression (CQR) [26] to find the prediction interval $C({\overset{ˆ}{Y}}^{n_{p}+1})$ satisfying the conformal coverage guarantee [33]:

(1) $$P[Y^{n_{p}+1}\in C({\overset{ˆ}{Y}}^{n_{p}+1})]\geq 1-\alpha$$

where α is a user-specified miscoverage rate. We attain $C({\overset{ˆ}{Y}}^{n_{p}+1})$ by adjusting the upper and lower bounds of ${\overset{ˆ}{Y}}^{n_{p}+1}$ with an offset q that is computed from the calibration dataset to satisfy (1):

(2) $$C({\overset{ˆ}{Y}}^{n_{p}+1})=[Q_{\alpha /2}({\overset{ˆ}{Y}}^{n_{p}+1})-q,Q_{1-\alpha /2}({\overset{ˆ}{Y}}^{n_{p}+1})+q]$$

where $Q_{\alpha }(.)$ is the function that estimates the αth quantile.^5^ Finally, we retrieve the volumes 1) closest to the upper and lower bounds of the prediction intervals $\left[{\overset{ˆ}{V}}_{LB}^{n_{p}+1},{\overset{ˆ}{V}}_{UB}^{n_{p}+1}\right]$ based on the $L_{1}$ norm, 2) contained within the prediction intervals (inliers), and 3) outside the prediction intervals (outliers). We provide an overview in Fig. 1 and pseudo-code in Algorithm 1.

Similar to prior work [4], we use sample quantiles instead of regression quantiles. Our method can be interpreted as a discrete version of CQR that finds marginal prediction intervals for downstream metrics given a patient. Our method is different to prior work in pixel-wise [3] that produce prediction intervals per reconstruction. Instead, we provide prediction sets directly from a set of patient reconstructions where each patient has different reconstruction volume sizes. We compare our method with conventional pixel-wise bounds.

**Algorithm 1:** Metric-guided Image Reconstruction Bounds

▷ Perform calibration to get upper and lower bound adjustment using CQR
**for** $i=1:n_{p}$ **do**
${\mathrm{score}}_{i}=max[Q_{\alpha /2}({\overset{ˆ}{Y}}^{i})-Y^{i},Y^{i}-Q_{1-\alpha /2}({\overset{ˆ}{Y}}^{i})]$
**end for**
$q=Q_{\frac{\left\lceil \left(n_{p}+1\right)(1-\alpha )\right\rceil }{n_{p}}}(\mathrm{scores})$
▷ Compute prediction interval for patient in test dataset
$C({\overset{ˆ}{Y}}^{n_{p}+1})=[LB({\overset{ˆ}{Y}}^{n_{p}+1}),UB({\overset{ˆ}{Y}}^{n_{p}+1})]=[Q_{\alpha /2}({\overset{ˆ}{Y}}^{n_{p}+1})-q,Q_{1-\alpha /2}({\overset{ˆ}{Y}}^{n_{p}+1})+q]$
▷ Retrieve upper and lower bound reconstructions
${\overset{ˆ}{V}}_{LB}^{n_{p}+1}\;=\arg{min}_{{\overset{ˆ}{V}}_{j}^{n_{p+1}}}\left|{\overset{ˆ}{Y}}_{p}^{n_{p}+1}-LB\left({\overset{ˆ}{Y}}^{n_{p}+1}\right)\right|$
${\overset{ˆ}{V}}_{UB}^{n_{p}+1}=\arg{min}_{{\overset{ˆ}{V}}_{j}^{n_{p+1}}}\left|{\overset{ˆ}{Y}}_{j}^{n_{p}+1}-UB\left({\overset{ˆ}{Y}}^{n_{p}+1}\right)\right|$
▷ Retrieve inliers and outlier reconstructions
**for** $j=1:n_{r}$ **do**
**if** ${\overset{ˆ}{Y}}_{j}^{n_{p}+1}\in [LB({\overset{ˆ}{Y}}^{n_{p}+1}),UB({\overset{ˆ}{Y}}^{n_{p}+1})]$ **then**
Add ${\overset{ˆ}{Y}}_{j}^{n_{p}+1}$ to inliers
**else**
Add ${\overset{ˆ}{Y}}_{j}^{n_{p}+1}$ to outliers
**end if**
**end for**

## 3 Experiments

### Radiotherapy Planning:

We use the Radiation Planning Assistant (RPA, FDA 510(k) cleared), a web-based tool for radiotherapy planning. [1,8,18]. RPA automates treatment planning on CT images and provides dose and plan reports for clinics in low-and-middle-income countries [1,8,18]. Dose statistics specify what percentage of organ volume receives a particular dose. Structural statistics are from organ segmentation and specify metrics such as organ volume and Hausdorff distance [15]. We use a dose prescription of 25 fractions in 50Gy (2.00Gy/fraction) for supraclavicular (SCV) and tangential field irradiation. The RPA automatically segments organs at risk and then applies a single-isocenter technique with matched tangential and SCV fields to treat the chest wall and SCV region.

### Dataset:

We use a de-identified CT dataset of 20 patients retrospectively treated with radiotherapy at The University of Texas MD Anderson Cancer Center. All CT images were of patients who had received surgical mastectomy to the right side of the body, and radiotherapy to the post-mastectomy chest wall and/or axillary lymph nodes. This research was conducted using an approved institutional review board protocol. Each ground truth CT is of size (512 × 512 × Number of slices). For each patient, we generate 10 digitally reconstructed radiographs (DRR) from the ground truth CT scan using the TIGRE toolbox [5]. The DRRs simulate image acquisition from a cone-beam geometry. We simulate physical randomness (beam angle variability and sensor noise) by generating DRRs with 3% noise and 50 random projections between 0 and 360 degrees. The number of projections was increased from 2 to 50 until organ boundaries were perceptually discernible in the reconstruction by the RPA. Because this work aims to showcase the feasibility of CP for image reconstruction, we assume that such a low-cost sv-CT device will be created in future work that gives acceptable reconstruction image quality. We use a self-supervised model, Neural Attenuation Fields (NAF), for reconstruction [35]. Each reconstruction is uncropped and contains the full scan. We use the default parameter setting in NAF [35] and introduce computational randomness through random initializations of NAF [30,19]. Ultimately, we construct a dataset of 20 patients with 10 reconstructions each. To construct the conventional pixel-wise upper and lower bounds, we take each pixel’s upper and lower quantiles.

### 3.1 Validation

We validate our method by computing coverage (Table 1), which is defined as the fraction of patients with ground truth metrics within the bounds. For metric-guided bounds, we use leave-one-out cross-validation on 20 patients and report the average coverage for metrics volume of ipsilateral lung that received 20Gy (Right Lung $V_{20}$), maxmimum dose to the heart (Heart $D_{0}$), and dose that 35% volume of the ipsilateral lung receives (Right Lung $D_{35}$). For conventional pixel-wise bounds, we compute the coverage of all patients. We use the finite sample correction $(1-\alpha {)}_{\mathrm{adj}}=\frac{\left\lceil \left(n_{p}+1\right)(1-\alpha )\right\rceil }{n_{p}}$ [26,2] for target coverage of $[(1-\alpha {)}_{\mathrm{adj}}]\%$. Our results show that metric-guided bounds give valid coverages for downstream tasks while conventional pixel-wise bounds do not.

### 3.2 Upper and lower bound retrieval

**Fig.2.**
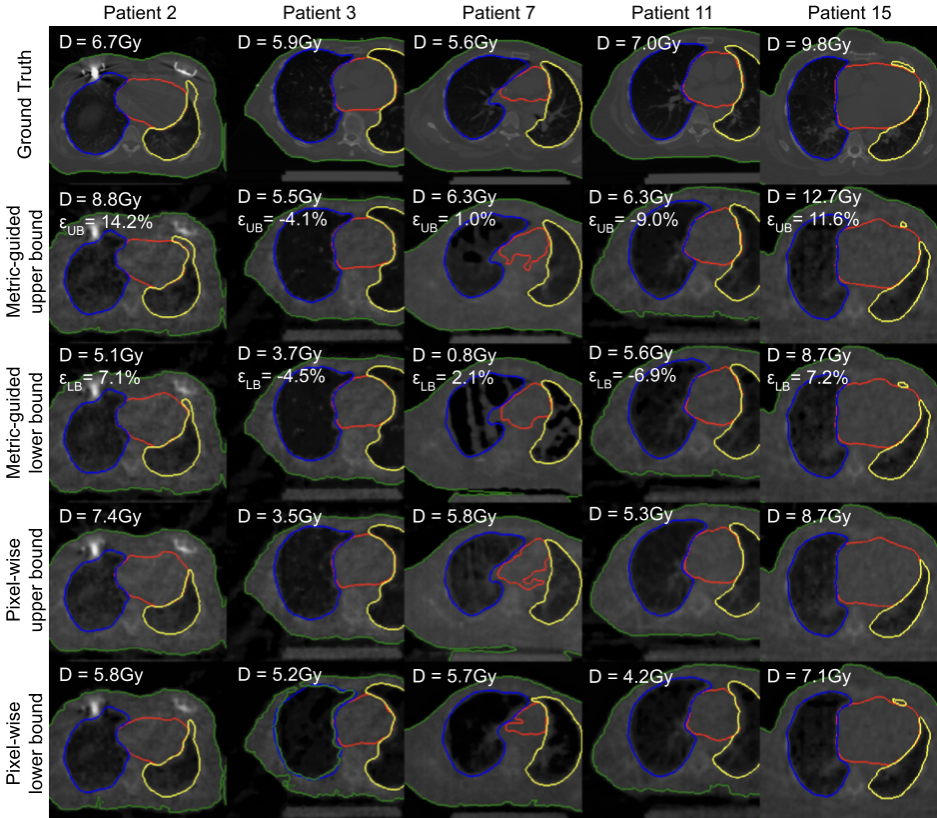
Metric-guided bounds account for spatial correlations that affect downstream metrics. For maximum dose to the heart (D) with target coverage of 90%, we show contours for heart (red), right lung (blue), left lung (yellow), and body (green) overlaid on CT slices. Pixel-wise upper and lower bounds differ in pixel-wise intensity, while metric-guided bounds differ in the spatial distribution of pixel intensities. Pixel-wise upper bounds have larger heart volumes than lower bounds, while metric-guided bounds have similar heart volumes. Retrieval error $\varepsilon_{B}$ is the difference between estimated and actual bound divided by the interval length.

We retrieve metric-guided and pixel-wise upper and lower bounds for a target coverage of 90% for maximum dose to the heart (Fig. 2). To verify the retrieved images are representative of the bounds at test time, we compute retrieval error defined as:

(3) $$\varepsilon_{B}=\frac{{\overset{ˆ}{Y}}_{B}^{n_{p}+1}-B^{n_{p}+1}}{UB^{n_{p}+1}-LB^{n_{p}+1}}\times 100\%$$

where B denotes the calibrated bound and can be upper bound UB or lower bound LB, and ${\overset{ˆ}{Y}}_{B}^{n_{p}+1}=arg\;{min}_{{\overset{ˆ}{Y}}_{j}^{n_{p}+1}}\left|{\overset{ˆ}{Y}}_{j}^{n_{p}+1}-B^{n_{p}+1}\right|$ are the estimated metrics closest to the calibrated bounds. We find that pixel-wise upper and lower bounds are perceptually similar and only differ in their intensity, while metric-guided bounds differ in the spatial distribution of pixel intensities. This indicates that metric-guided bounds take spatial correlations into account. As a consequence, the pixel-wise differences for metric-guided bounds can be both positive and negative. This indicates that single pixels do not carry sufficient information to explain the variations in dose. We find that the segmentations of the heart are also perceptually different. Pixel-wise upper bounds tend to have larger volumes than lower bounds, while this rule does not hold for metric-guided bounds.

Furthermore, this result suggests that pixel-wise and metric-guided methods may disagree on inliers and outliers. Metric-guided inlier reconstructions may have pixels considered as pixel-wise outliers and metric-guided outlier reconstructions may have pixels considered as pixel-wise inliers.

### 3.3 Anatomical Differences

**Fig.3.**
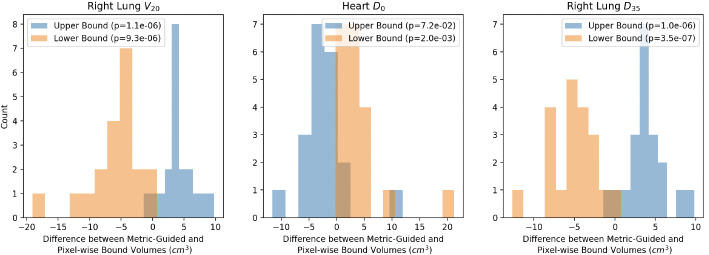
Metric-guided and Pixel-wise methods produce anatomically different upper and lower bounds. We determine whether the upper and lower bound volumes from metric-guided and pixel-wise methods are different across methods using paired t-tests. For all three metrics - volume of ipsilateral lung that received 20Gy (Right Lung $V_{20}$), maximum dose to the heart (Heart $D_{0}$) and dose that 35% volume of the ipsilateral lung receives (Right Lung $D_{35}$), we find that the differences are significant (p<0.05) except for the upper bound reconstructions for Heart $D_{0}$.

Using organ segmentations from RPA, we determine whether there is a statistically significant difference in upper bound volume across methods and lower bound volume across methods using paired t-tests. We use the dose metrics in Table 1. We find statistically significant differences (p<0.05) for upper and lower bounds across methods except for the upper bound reconstructions for Heart $D_{0}$ (p=7.2e-2) (Fig. 3). This suggests that the upper and lower bounds across methods are anatomically different.

## 4 Conclusion

We propose a method that leverages conformal prediction to retrieve upper/lower bounds and statistical inliers/outliers of reconstructions based on the prediction intervals of downstream metrics. We apply our method to sv-CT for downstream radiotherapy planning and show 1) metric-guided bounds have valid coverage for downstream metrics unlike conventional pixel-wise bounds and 2) statistically significant anatomical differences of upper/lower bounds between metric-guided and pixel-wise methods.

## 5 Discussion

There are several areas for further investigation:

### Factors affecting retrieval error.

Retrieval error may depend on number of samples, the diversity of samples, and the accuracy of the model. The prediction intervals and retrieval errors may also be very large if the model is highly biased. Asymmetric bounds could help identify this bias [26]. Furthermore, we assume the downstream processes to be deterministic. This is an appropriate assumption for the maximum dose to the heart, but may not be for other parameters. Opportunities lie in decoupling uncertainty from physical, reconstruction algorithm, and downstream algorithm randomness [13].

### Evaluating Safety and Equity.

We can perform patient-specific safety evaluations and identify inequities across patients. For a dose prescription of 50Gy (2Gy/fraction), a safe maximum dose to the heart is <5Gy and the volume of the ipsilateral lung getting 20Gy is <35%. If the upper bound of the prediction interval is greater than these thresholds, it may indicate that the reconstruction is unsuitable for planning. Patients or measurement conditions with high uncertainty can be used for downstream interpretation [25,22] and action [34,20]. They may correspond to specific clinical scenarios, such as inadequately filled lungs or large distance from heart to chest wall. Opportunities lie in applying causal methods [24,28,23] to identify factors causes of high uncertainty.

### Test time evaluation metrics for reconstruction.

While we show inliers and outliers for one metric, our method can be extended to multiple metrics [21,31] where we find reconstructions with all estimated metrics in the prediction intervals containing the ground truth metrics with confidence. Opportunities lie in assessing reconstructions with multiple critical metrics.

### Other applications.

Opportunities lie in extending our method to other medical imaging applications [22,17] and critical scenarios. Additionally, although not demonstrated in our work, our method does not necessitate reconstruction samples to be of identical size or dimensions, as calibration is conducted based on a scalar downstream metric.

## Figures and Tables

**Table 1.** Metric-guided bounds yield valid coverages while conventional pixel-wise bounds do not. Using 20 patients and target coverage of 90%, we perform leave-oneout cross-validation and compute average coverage using metric-guided and pixel-wise methods for maximum dose to the heart (Heart $D_{0}$), volume of ipsilateral lung that received 20Gy (Right Lung $V_{20}$), volume of ipsilateral lung (Right Lung Volume), and dose that 35% volume of the ipsilateral lung receives (Right Lung $D_{35}$).

Method	Heart $D_{0}$	Right Lung Volume	Right Lung $V_{20}$	Right Lung $D_{35}$
Metric-guided	90	90	90	90
Pixel-wise	75	0	50	50

## Footnotes

3 While we concentrate on imaging, our method can be applied to any multidimensional setting.

4 We do not have ground truth volume $V^{n_{p}+1}$ and metric $Y^{n_{p}+1}$ at test time.

5 While we use symmetric adjustments in this work, asymmetric bounds can also be used. See [26] for more details.
